# Whole-genome sequencing reveals adaptations of hairy-footed jerboas (*Dipus*, Dipodidae) to diverse desert environments

**DOI:** 10.1186/s12915-023-01680-5

**Published:** 2023-08-30

**Authors:** Xingwen Peng, Jilong Cheng, Hong Li, Anderson Feijó, Lin Xia, Deyan Ge, Zhixin Wen, Qisen Yang

**Affiliations:** 1 Key Laboratory of Zoological Systematics and Evolution, Institute of Zoology, Chinese Academy of Sciences, Chaoyang District, Beijing, 100101 China; 2 University of Chinese Academy of Sciences, Shijingshan District, Beijing, 100049 China; 3 Novogene Bioinformatics Institute, Haidian District, Beijing, 100083 China; 4 Negaunee Integrative Research Center, Field Museum of Natural History, Chicago, IL 60605 USA

**Keywords:** *Dipus*, High-altitude adaptation, Hyper-arid adaptation, Energy homeostasis, Water balance, Population genomics

## Abstract

**Background:**

Environmental conditions vary among deserts across the world, spanning from hyper-arid to high-elevation deserts. However, prior genomic studies on desert adaptation have focused on desert and non-desert comparisons overlooking the complexity of conditions within deserts. Focusing on the adaptation mechanisms to diverse desert environments will advance our understanding of how species adapt to extreme desert environments. The hairy-footed jerboas are well adapted to diverse desert environments, inhabiting high-altitude arid regions, hyper-arid deserts, and semi-deserts, but the genetic basis of their adaptation to different deserts remains unknown.

**Results:**

Here, we sequenced the whole genome of 83 hairy-footed jerboas from distinct desert zones in China to assess how they responded under contrasting conditions. Population genomics analyses reveal the existence of three species in hairy-footed jerboas distributed in China: *Dipus deasyi*, *Dipus sagitta*, and *Dipus sowerbyi*. Analyses of selection between high-altitude desert (elevation ≥ 3000m) and low-altitude desert (< 500m) populations identified two strongly selected genes, *ATR* and *HIF1AN*, associated with intense UV radiation and hypoxia in high-altitude environments. A number of candidate genes involved in energy and water homeostasis were detected in the comparative genomic analyses of hyper-arid desert (average annual precipitation < 70mm) and arid desert (< 200mm) populations versus semi-desert (> 360mm) populations. Hyper-arid desert animals also exhibited stronger adaptive selection in energy homeostasis, suggesting water and resource scarcity may be the main drivers of desert adaptation in hairy-footed jerboas.

**Conclusions:**

Our study challenges the view of deserts as homogeneous environments and shows that distinct genomic adaptations can be found among desert animals depending on their habitats.

**Supplementary Information:**

The online version contains supplementary material available at 10.1186/s12915-023-01680-5.

## Background

Since Darwin’s era, biologists have been fascinated by the adaptation of organisms to their environments, especially hostile ones. Deserts cover more than one third of the land surface area, and their extreme environmental conditions, aridity, lack of resources, extreme thermal amplitude, and intense radiation make them a natural laboratory for studying adaptation of organisms to extreme habitats [1]. Evolutionary ecology researches have revealed a variety of behavioral, anatomical, and physiological adaptations in desert species, but the genetic basis of these adaptations is still limited [1–3]. Studying the genetic basis of desert adaptation will strengthen our understanding of the role of past climatic processes [e.g., desertification] in driving adaptation, assess the potential impacts of current climate change on living organisms, and thus facilitate efforts to save biodiversity currently threatened by increased desertification.

Genomic studies of different desert mammals have provided some evidence for the genomic basis of desert adaptation and found that multiple desert species exhibit convergent evolution at the genetic level involving the phenotype of energy and water homeostasis [3]. For instance, genes related to salt metabolism are under convergent positive selection in large desert ungulates [4–6], while genes associated with fat metabolism, insulin signaling pathways, and water retention are under convergent positive selection in both large desert ungulates and small desert rodents [4, 5, 7–10]. Most of genome studies on desert adaptations, however, are based on comparisons between desert and non-desert species, overlooking the diversity of environment conditions within the desert biome related to distinct geological origins and climatic conditions [1]. Intraspecific genomic comparisons among different desert environment populations could detect correlations between variations putatively under local environmental selection and specific climate variables, advancing our understanding of the genetic basis of species adaptation to deserts.

The hairy-footed jerboa (*Dipus*; Dipodidae) is a typical psammophilous rodent and has thrived in many types of deserts in Asia [11–14]. These deserts differ greatly in elevation and precipitation gradient due to their unique geological history, such as the tectonic uplift of the Qinghai-Tibetan Plateau and the retreat of the Paratethys Sea [15]. For example, the Qaidam Desert is the highest non-polar desert in the world with an average elevation around 3000m, and the Taklimakan Desert located in the westernmost portion of China is the driest (mean annual precipitation < 50mm), while sandy lands in the east are much more humid [16, 17]. Different environmental pressures promote different evolutionary processes in genomic regions, which may lead to heterogeneous patterns of genomic differentiation among different desert populations of hairy-footed jerboas. Thus, these animals provide an excellent model to gain insights into genetic mechanisms underlying the adaptations of mammals to diverse desert environments.

**Fig. 1:**
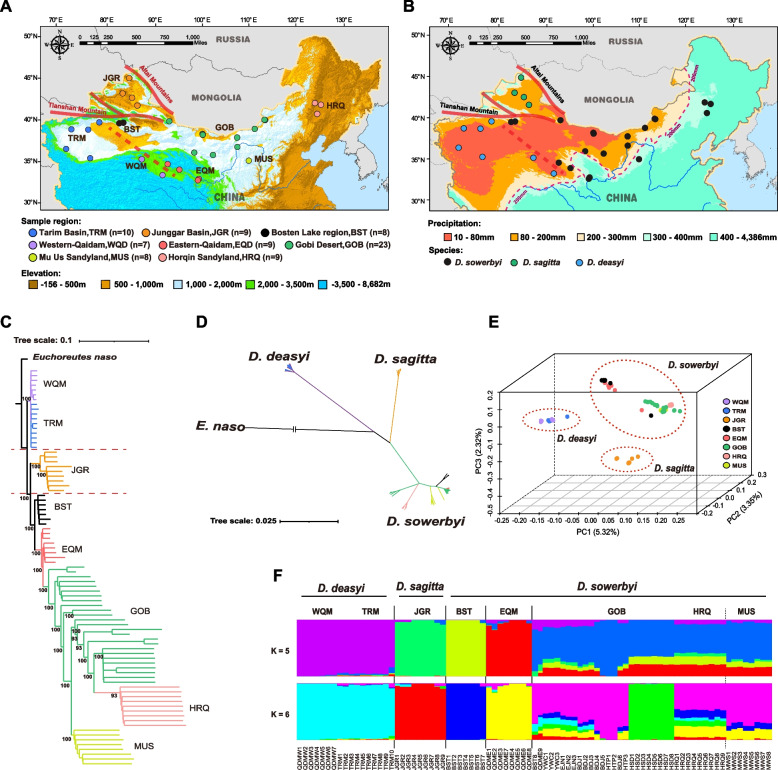
Geographic distribution and population genetic analyses of *Dipus* jerboas in deserts of China. **A** Sampling sites of 83 *Dipus* jerboas in China desert region covering 8 geographical populations. The elevation (m) of the study area is also visualized. **B** Geographic variation of the annual mean precipitation (mm). Precipitation data from 1961 to 2019 were downloaded from the WorldClim database (https://www.worldclim.org/, last accessed March 18, 2022). The 200 mm average annual precipitation line is also visualized. **C** Neighbor-joining (NJ) tree based on whole-genome SNPs using *p-*distances between individuals. The long-eared jerboa, *Euchoreutes naso*, is used as an outgroup. The number beside the tree nodes indicates the bootstrap value. **D** NJ tree based on the mitochondrial genome of all 83 *Dipus* individuals. **E** Principal components analysis of 83 *Dipus* individuals based on whole-genome SNPs. **F** Population genetic structure of the 83 *Dipus* jerboas inferred from the program sNMF v.1.2. The length of each color segment represents the proportion of the individual genome inferred from ancestral populations (*K*= 5-6, which had a low cross-validation error). See supplementary Table s1, Supplementary Material online for the abbreviations of the geographical populations and individuals

Herein, we sequenced the whole genome of 83 hairy-footed jerboas from different desert environments in China. Given the uncertainty taxonomy classification within hairy-footed jerboas [11, 12, 18, 19], we first reassessed the phylogenetic status of these 83 individuals based on genomic data to reduce the impact of interspecies genomic differences on subsequent genome-wide selection sweeps analyses. A comprehensive analysis of population structure, demographic history, gene flow, and species delimitation revealed that the hairy-footed jerboas in China contain three species: *Dipus deasyi* Barret-Hamilton, 1900; *Dipus sagitta* Pallas, 1773; and *Dipus sowerbyi* Thomas, 1908. Among them, *D. sowerbyi* is a habitat generalist, inhabiting the Qaidam Basin (defined as ‘high-altitude desert’, altitude ≥ 3000m), the Horqin Sandy Land (defined as ‘low-altitude desert’, altitude < 500m), hyper-arid desert region (average annual precipitation < 70mm; [20]), arid desert region (< 200mm), and semi-desert zone (> 360mm; Fig. 1A and B, Additional file 1: Tables s1 and s2). Through comparing the genomes of jerboa populations from relatively extreme environments and contrasting desert conditions, we revealed distinct adaptive genetic mechanisms to diverse desert environments.

## Results

### Whole-genome sequencing and SNP calling

To better understand the phylogenetic relationships and evolutionary history of the member of hairy-footed jerboas in China’s arid region, we sequenced 83 samples from eight areas, including Horqin Sandy Land (HRQ, *n* = 9), Gobi Desert (GOB, *n* = 23), Mu Us Desert (MUS, *n* = 8), Bosten Lake region (BST, *n* = 8), the eastern of Qaidam Basin (EQM, *n* = 9), the western of Qaidam Basin (WQM, *n* = 7), Junggar Basin (JGR, *n* = 9), and Tarim Basin (TRM, *n* =10), covering almost all of their representative habitats in China (Fig. 1A, B; Additional file 1: Table s1). We also sequenced a long-eared jerboa (*Euchoreutes naso*, Euchoreutinae, Dipodidae) as phylogenetic outgroup. The 83 individuals yielded a total of 17.25 Tb of Illumina clean reads and 5125.96 Gb high-quality genomic data. The average sequence depth and coverage per individual were 12.07× (6.49-31.77×) and 97.11% (95.39-98.64%) (see Additional file 1: Table s3). Moreover, we successfully assembled the 84 jerboas’ mitochondrial genome sequences from their genome resequencing reads (see Additional file 2).

Using the hairy-footed jerboa reference genome (NCBI accession number: PRJNA682294; representing a *D. sowerbyi* according to our new classification) generated in our previous study [10, 21] for single nucleotide polymorphism (SNP) calling and strict quality filtering, we obtain 25.36 million high-quality SNPs and 2.60 million InDels for all 83 hairy-footed jerboas. SnpEff v4.3 [22] annotation showed that most of SNPs were located in intergenic regions (18.73 million, 68.07%) and 31.39% in or near genes (8.63 million). Only 137,397 SNPs (0.50%) were located in protein-coding exons, causing 64,733 nonsynonymous and 70,362 synonymous mutations, with 3871 start codon and stop codon changes, showing their potential impact on function of relevant genes (Additional file 1: Table s4). The nonsynonymous/synonymous ratio was 0.92. The similar pattern was observed in InDels annotation result with most of variants in intergenic regions and in or near genes (Additional file 1: Table s4).

### Phylogeny and population structure of hairy-footed jerboas in China

Based on the genome-wide SNP data and assembled mitochondrial genome data of all hairy-footed jerboas and one long-eared jerboa as outgroup, we first clustered individuals using phylogenetic reconstruction analysis. Both neighbor-joining (NJ) trees based on whole-genome SNPs and mitochondrial genomes revealed strong clustering of hairy-footed jerboas into three distinct genetic lineages (mean* F*_ST_ = 0.1008–0.2722; Additional file 1: Table s5): TRM-WQM (referred to as *D. deasyi* here;* n* = 17), JGR (referred to as *D. sagitta* here; *n* = 9), and HRQ-GOB-MUS-BST-EQM (referred to as *D. sowerbyi* here; *n* = 57; Fig. 1C, D; Additional file 3: Fig. s1 and s2). A principal component analysis (PCA) and a sparse non-negative matrix factorization algorithm-based genetic clustering analysis (using sNMF method) also recapitulated these groupings (Fig. 1E, F; Additional file 3: Fig. s3 and s4). In summary, our results revealed three major evolutionarily significant units of hairy-footed jerboas in China’s arid region, reflecting the three *Dipus* species recognized here, and are generally consistent with previous taxonomic assessment [12]. The distribution of these three species is well delimitated by clear geographic barriers (Fig. 1A, B, and F, Additional file 3: Fig, s4). Importantly, we observed multiple ancestral sub-lineages in *D. sowerbyi*, which reflect marked ancestral polymorphism in this species (Fig. 1 F). For example, the ancestry components number *K* = 5 or 6 had the lower cross-validation error in the clustering analysis (Additional file 3: Fig. s5A), which represented two optimal ancestor component composition patterns. Nevertheless, genetic clustering analysis for the 57 samples of *D. sowerbyi* showed that *K* = 1 had the lowest cross-validation error (Additional file 3: Fig. s5B), supporting the recognition of only one species in this group.

### Demographic history and species delimitation

**Fig. 2:**
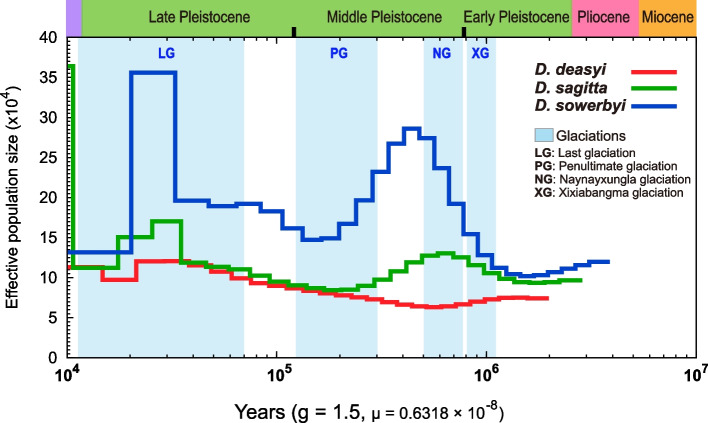
Demographic history of three *Dipus* species of China. PSMC results for the representative individuals with high read coverage show different demographic histories of the three *Dipus* species with a generation time (g) of 1.5 years and a mutation rate (μ) of 6.319× 10^−9^ per site per generation

Pairwise sequential Markovian coalescent (PSMC) analysis of individuals with the highest coverage in each lineage showed that the demographic histories of hairy-footed jerboas could be traced back to approximately 3.8 Mya (million years ago; Pliocene), and the effective population sizes (*N*_e_) trajectories of the three lineages were highly inconsistent (Fig. 2). The *N*_e_ curves of the three lineages split at ~2–1 Mya (Fig. 2), possibly suggesting a fairly ancient divergence of the three species and is consistent with the geographical isolation effect caused by the orogenic movement in western China at that time [23, 24]. During the early Pleistocene (~2.58Mya), the Tianshan and Altai mountains had already reached their present heights [23, 24]. The barrier effect of the Tianshan and Altai mountains increased the aridification of local climate and may have hindered the contact among these three lineages, facilitating their independent evolution. Regardless of the *N*_e_ size, *D. sagitta* and *D. sowerbyi* had the same trend in curve trajectory throughout the Pleistocene, with two apparent expansions and two severe bottlenecks (Fig. 2). Their ancestral population first expanded and reached the *N*_e_ peak (~0.7 Mya for *D. sagitta*; ~0.5 Mya for *D. sowerbyi*; Fig. 2) during the Xixiabangma glaciation (1.17–0.8 Mya) and Naynayxungla glaciation (0.78–0.5 Mya), and the latter was the most extensive glaciation in the Quaternary [25–27]. The dry-cold climate and the strengthening of winter monsoon at this ice age could have facilitated population expansion because an increase in sandy land and desert environment were expected under such climate conditions [28–33]. After the retreat of the Naynayxungla glaciation, the ancestral population of *D. sagitta* and *D. sowerbyi* both experienced a severe decline and the population bottleneck occurred ~0.2–0.17 Mya (Fig. 2). At that time, the climate was still cold but relatively wetter, which may have accelerated the expansion of grassland and the retreat of sandy land and deserts [33, 34]. Subsequently, their ancestral population size peaked again (~0.035 Mya; Fig. 2) during the Last glaciation because the dry-cold climate of glaciation intensified the expansion of the desert environment in China and connected it into a whole [33]. We also observed that *D. sowerbyi* showed significantly higher *N*_e_ than *D. sagitta* and *D. deasyi* throughout the Pleistocene and the maximum *N*_e_ difference between *D. sowerbyi* and *D. sagitta* and *D. deasyi* was at ~ 0.02–0.035 Mya (Fig. 2).

In addition, analyses using f3-statistics [35] and gene flow analysis [35, 36] indicated that none of the three hairy-footed jerboa lineages had any ancient admixture from other two lineages (f3-statistic = 0.003-0.027, *Z*-scores > 82.622; Additional file 1: Table s6 and Additional file 3: Fig. s6). Species delimitation analysis using the SNAPP method [37, 38] also supported complete reproductive isolation of three hairy-footed jerboa lineages, which provided strong statistical support for a “three-species model” (marginal likelihood estimate = −1.82 × 10^5^) instead of the previous “two-species model” classification (marginal likelihood estimate = −1.84 × 10^5^; Additional file 1: Table s7).

### Genetic adaptation to high-altitude desert environment of hairy-footed jerboa

**Fig. 3:**
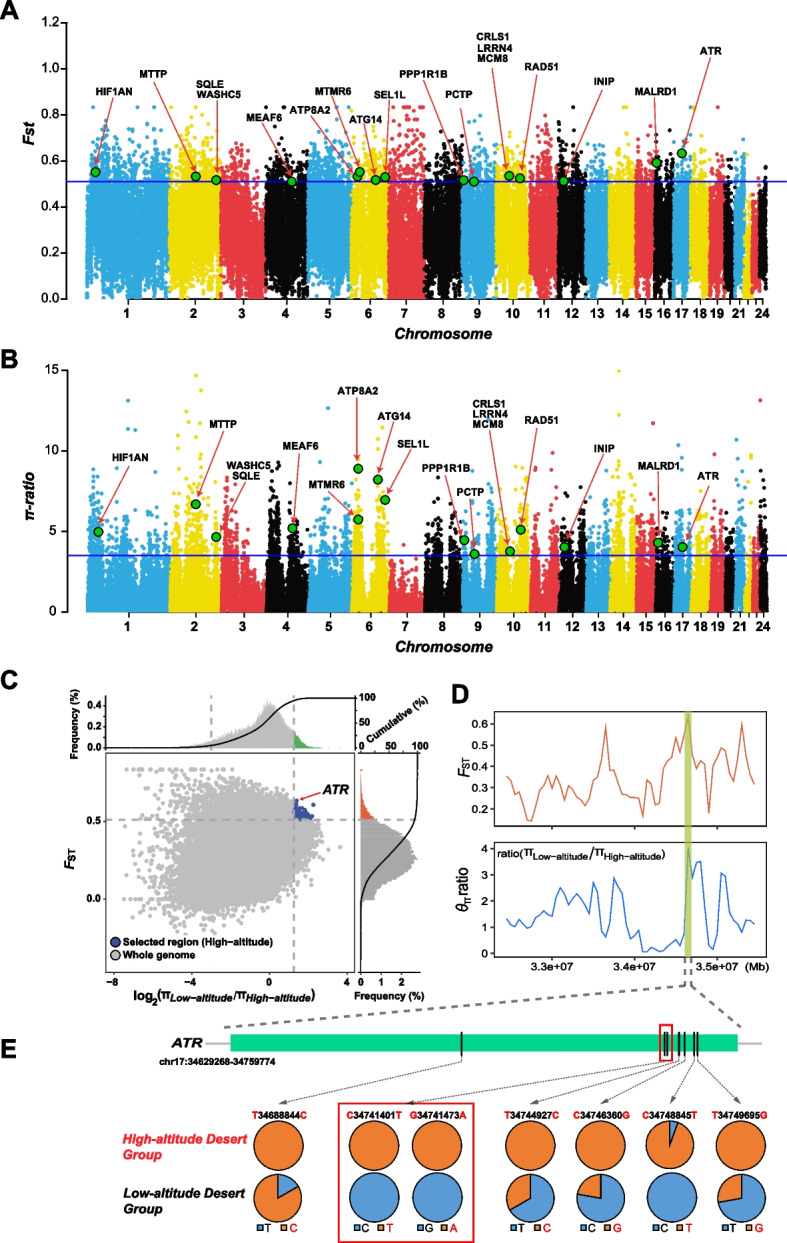
Genomic regions with strong selective signals in the genome of *Dipus sowerbyi* from the high-altitude desert environment. Distribution of the **A** fixation indices (*F*_ST_) and **B** *π*-ratio values calculated in 100-kb sliding windows with 50-kb steps. The horizontal blue lines above the figure represent the top 5% threshold with **A** *F*_ST_ > 0.51 and **B** *π*-ratio > 3.51. The candidate genes shown in** A** and** B** are closely related to high-altitude desert adaptation, with functions related to radiation response, hypoxia, DNA repair, and lipid homeostasis. **C** Distribution of log_2_(π-ratios) and *F*_ST_ values calculated in 100-kb sliding windows with 50-kb increments between high-altitude desert group and the control low-altitude desert group. Data points in blue (corresponding to the top 5% of the empirical log_2_(π-ratios) values and the top 5% of the empirical* F*_ST_ values) are genomic regions under selection in the high-altitude desert group. **D** Log_2_(π-ratios) and *F*_ST_ values were calculated using VCFtools for each 50-kb sliding window with 25-kb increment around the gene *ATR*. **E** Allele frequencies of seven missense mutations within the ATR gene between the high-altitude desert group and the low-altitude desert group

*Dipus sowerbyi* inhabits the high-altitude desert regions (altitude ≥ 3000m) on the eastern side of the Qinghai-Tibetan Plateau, which are much higher than other desert environments, such as the Horqin Sandy Land (altitude < 500m) in the eastern China (Fig. 1A, Additional file 1: Table s1). To identify candidate mutations that may have been under positive selection specifically in the populations at high-altitude arid environments, we combined *F*_ST_ and *π*-ratios analysis with 100-kb sliding window and shift of 50 kb (low-altitude desert group versus high-altitude desert group; Additional file 1: Table s2) to scan for footprints of positive selection in genomic regions. Using the top 5% of *F*_ST_ and *π*-ratio cutoffs, we identified 88 candidate genes with strong selection signals from the high-altitude desert group (Fig. 3A–C, Additional file 1: Table s8).

Gene ontology (GO) analyses revealed that 17 candidate genes (*ATG14*, *ATP8A2*, *ATR*, *CRLS1*, *INIP*, *LRRN4*, *MALRD1*, *MCM8*, *MEAF6*, *MTTP*, *PCTP*, *PPP1R1B*, *RAD51*, *SEL1L*, *SQLE*, *TMTR6*, and *WASHC5*; Fig. 3A, B) were significantly over-represented by functions related to radiation response (GO:009314, response to radiation; GO:0010212, response to ionizing radiation; and GO:009416, response to light stimulus; *P* < 0.05), DNA repair (GO:0000724, double-strand break repair via homologous recombination; GO:0000725, recombinational repair; GO:2000779, regulation of double-strand break repair; GO:0006302, double-strand break repair; *P* < 0.05), and lipid homeostasis and metabolism (GO:0055088, lipid homeostasis; GO:0055092, sterol homeostasis; GO:0016125, sterol metabolic process; GO:0046486, glycerolipid metabolic process; GO:1902652, secondary alcohol metabolic process; *P* < 0.05; see Additional file 1: Table s9), which were directly related to increased UV radiation (UV), hypoxia, and scarce food resources at high-altitude desert. One of them, *ATR*, exhibited the highest *F*_ST_ and higher π-ratio value (Fig. 3C, D), indicating that a strong selective sweep occurred in this gene. *ATR* encodes a serine/threonine protein kinase, which is a key protein that acts as a DNA damage sensor that activates checkpoints signaling in response to genotoxic stress such as ionizing radiation, UV, or DNA replication arrest, and promotes DNA repair, recombination, and apoptosis [39–41]. Within the *ATR* gene, seven missense mutations in the high-altitude desert group had significantly higher frequencies than those observed in the low-altitude desert group, especially two of the missense mutations (chr17: 34741401, C > T; chr17: 34741473, G > A) were fixed in the high-altitude desert group but remained invariant in the low-altitude desert group (Fig. 3E, Additional file 1: Table s10). Mutations in the coding sequence cause alterations in amino acids that will lead to changes in protein structure and function. This may reflect the importance of UV response in the adaptation of hairy-footed jerboas to high-altitude desert environments.

Another candidate gene, *HIF1AN*, ranking within the top 20 *F*_ST_ value (Additional file 1: Table s8), was functionally involved in response to hypoxia based on the previous functional studies [42–44]. The hypoxia-inducible factor (HIF)-mediated transcriptional response pathway, also known as the HIF-1 signaling pathway, is a classic pathway for mammalian cells to adapt to hypoxia conditions [43, 45]. *HIF1AN* (Hypoxia-inducible factor 1-alpha inhibitor) encodes an asparagine hydroxylase, which can interact with *HIF* and inhibit its transcriptional activity, thus regulating animal adaptation to anoxic environment [42–44]. Further examination of this candidate gene identified four nucleotides (chr1: 35350203, G > C; chr1: 35351699, T > C; chr1: 35352064, G >C; and chr1: 35354031, C > T) had mutated in high-altitude desert group but remained unchanged in the low-altitude desert group (Additional file 3: Fig. s7). Although these mutations occur in the intron region and do not directly affect the amino acid sequence of the protein, they may potentially affect gene expression or transcription and mRNA processing, thus affecting the interaction between *HIF1AN* and *HIF*. Therefore, the mutation in *HIF1AN* likely contributed to hypoxia adaptation of hairy-footed jerboas in high-altitude environments.

### Genetic adaptation to hyper-arid desert of hairy-footed jerboas

**Fig. 4:**
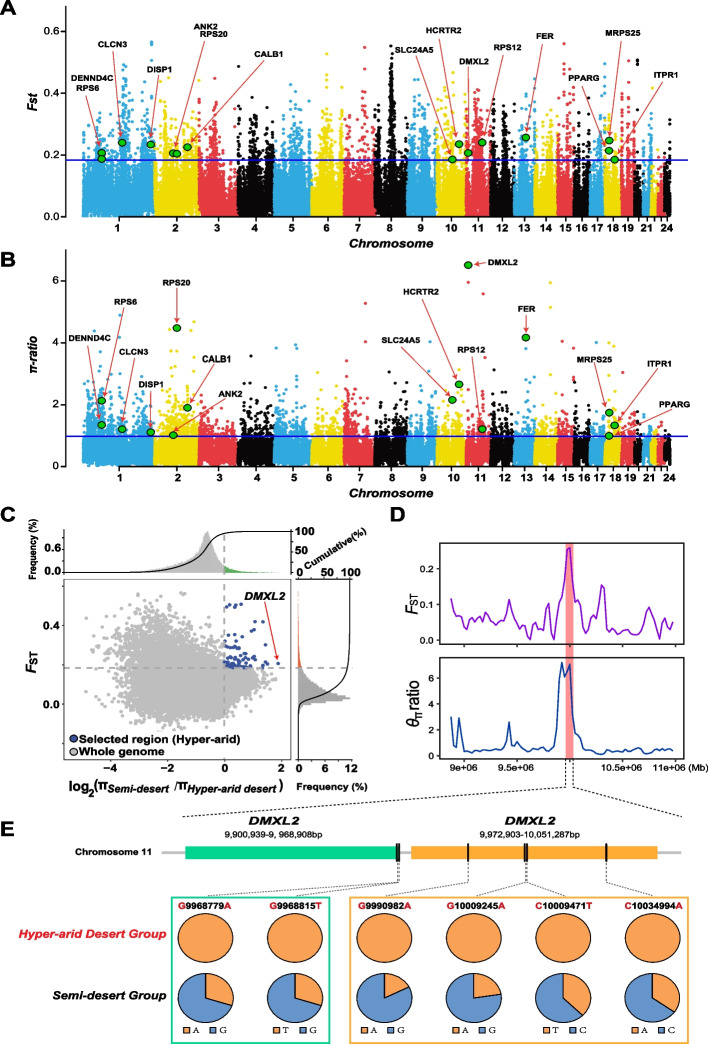
Genomic regions with strong selective signals in the genome of *Dipus sowerbyi* from the hyper-arid desert environment. Distribution of the **A** fixation indices (*F*_ST_) and **B** π-ratio values calculated in 100-kb sliding windows with 50-kb steps. The horizontal blue lines above the figure represent the top 5% threshold with **A **F_ST_ > 0.18 and **B** π-ratio > 0.98. The candidate genes shown in **A** and** B** are closely related to hyper-arid desert adaptation, with functions related to energy metabolism and cellular homeostasis. **C** Distribution of log_2_(π-ratios) and *F*_ST_ values calculated in 100-kb sliding windows with 50-kb increments between hyper-arid desert group and the control semi-desert group. Data points in blue (corresponding to the top 5% of the empirical log_2_(π-ratios) values and the top 5% of the empirical *F*_ST_ values) are genomic regions under selection in the hyper-arid desert group. **D** Log_2_(π-ratios) and *F*_ST_ values were calculated using VCFtools for each 50-kb sliding window with 25-kb increment around the gene *DMXL2*. **E** Allele frequencies of six missense mutations within the *DMXL2* gene between hyper-arid desert group and semi-desert group

Considering the large differences in the degree of aridity (Fig. 1B, Additional file 1: Table s1) between habitats inhabited by *D. sowerbyi* populations, we compared two relatively extreme populations (semi-desert group versus hyper-arid desert group; see Additional file 1: Table s2) in order to identify the genomic region responsible for jerboa adaptation to extreme arid environments. The overlap of the *F*_ST_ (top 5% outlier, *F*_ST_ > 0.184) and *π*-ratios analysis (top 5% outlier, *π*-ratio > 0.985) unveiled 43 positive selection genes (PSGs) in the hyper-arid desert group after annotation and removing repeats (Fig. 4A–C, Additional file 1: Table s11).

GO enrichment analyses showed that 15 PSGs (*ANK2*, *CALB1*, *CLCN3*, *DENND4C*, *DISP1*, *DMXL2*, *FER*, *HCRTR2*, *ITPR1*, *MRPS25*, *PPARG*, *RPS12*, *RPS20*, *RPS6*, and *SLC24A5*; Fig. 4A, B) were significantly enriched for biological processes associated with energy metabolism (GO:0042593, glucose homeostasis; GO:0032868, response to insulin; GO:0006518, peptide metabolic process; GO:0043043, peptide biosynthetic process; *P* < 0.05) and cellular homeostasis (GO:0019725, cellular homeostasis; GO:0050801, ion homeostasis; *P* < 0.05; Additional file 1: Table s12), indicating the importance of energy homeostasis and cellular chemical homeostasis for hairy-footed jerboas survival in hyper-arid desert with extremely scarce resources and strong osmotic stress. For example, the target gene *DMXL2* exhibited the largest π-ratio and higher *F*_ST_ value in the hyper-arid desert group, manifesting a strong selective sweep (Fig. 4C, D). *DMXL2* (DmX-like protein 2) encodes a protein with multiple WD40 domains and is involved in many cellular functions, including participating in regulation of insulin secretion and glucose homeostasis [46–49]. Knockout experiments in mice showed that loss of *DMXL2* function triggered severe hypoglycemia and death in neonates [46]. Further examination of this gene, we found six specific missense mutations with the allele frequency that differed greatly between hyper-arid desert group and semi-desert group (Fig. 4E, Additional file 1: Table s13). In particular, all alleles of six missense mutations were fixed in the hyper-arid desert group, whereas the allele frequency of the mutation allele was below 0.375 in the semi-desert group (Fig. 4E, Additional file 1: Table s12), suggesting that these specific advantageous alleles may be one of the key reasons for hairy-footed jerboas to survive in hyper-arid deserts.

Of the remaining 28 PSGs, six were also functionally involved in protein metabolism (*AASS* and *FBXL4*), lipid metabolism (*MSMO1*, *FADS2P1*, and *PLCD3*), and the tricarboxylic acid cycle (*SUCLG2*) based on the NCBI annotations and Swissport databases. This further suggested that selection in energy metabolism might be an important adaptive mechanism of hairy-footed jerboas under the hyper-arid conditions. Moreover, we found that three PSGs (*HPF1*, *NR2C2*, *SLC26A7*) were functionally associated with responding to multiple environmental stresses [50–53], coupled with strong ionic radiation and high osmotic stress in hyper-arid environments. For example, *SLC26A7* (solute carrier family 26 member 7), a member of the sulfate/anion transporter gene family, is specifically expressed in the kidney and plays a role in maintaining renal osmotic homeostasis by mediating the transport of bicarbonate, chloride, sulfate, and oxalate [52, 53].

### Genetic adaptation to arid desert environment of hairy-footed jerboas

To identify the genomic regions responsible for arid desert adaptation of hairy-footed jerboas, we compared 37 samples from the arid desert group (average annual precipitation < 200mm) and 20 samples from the semi-desert group in *D. sowerbyi* (> 360mm; see Additional file 1: Table s2). We found that 203 genes were both positively selected for the *F*_ST_ (top 5% outlier, *F*_ST_ > 0.102) and *π*-ratios analysis (top 5% outlier, *π*-ratio > 0.976) under the arid desert environment (Additional file 1: Table s14).

The functional enrichment analysis of PSGs identified 135 significantly over-represented (*P* < 0.05) biological process GO terms and 17 KEGG pathways (Additional file 1: Tables s15 and s16). Among them, 26 biological process GO terms and four KEGG pathways enriched by 52 PSGs were closely involved in adapting to arid desert. Specifically, four GO terms (GO:0030036, actin cytoskeleton organization; GO:0030029, actin filament-based process; GO:0007015, actin filament organization; GO:0030048, actin filament-based movement) and two KEGG pathways (mmu04924 Renin secretion; mmu04927: Cortisol synthesis and secretion) were associated with hyperosmotic stress. Four GO terms (GO:0000027, ribosomal large subunit assembly; GO:0042273, ribosomal large subunit biogenesis; GO:0042255, ribosome assembly; GO:0042254, ribosome biogenesis) and one KEGG pathway (mmu03010: Ribosome) were associated with ribosome. Three GO terms (GO:0006094, gluconeogenesis; GO:0019319, hexose biosynthetic process; GO:0046364, monosaccharide biosynthetic process) and two KEGG pathways (mmu04922: Glucagon signaling pathway; mmu04927: Cortisol synthesis and secretion) were associated with glucose homeostasis. Eight GO terms (GO:0043043, peptide biosynthetic process; GO:0006518, peptide metabolic process; GO:0043604, amide biosynthetic process; GO:0006412, translation; GO:0002181, cytoplasmic translation; GO:0034645, cellular macromolecule biosynthetic process; GO:0060193, positive regulation of lipase activity; GO:0060191, regulation of lipase activity) were associated with protein and lipid metabolism. Six GO terms (GO:2001020, regulation of response to DNA damage stimulus; GO:0080135, regulation of cellular response to stress; GO:0010569, regulation of double-strand break repair via homologous recombination; GO:2000779, regulation of double-strand break repair; GO:2001021, negative regulation of response to DNA damage stimulus; GO:0006281, DNA repair) were associated with stress response and DNA repair. Similarly, the phenotype enrichment analysis of these candidate genes in the arid desert group showed significant enrichments related to renal development, water retention, and increase energy intake. For instance, nine PSGs (*AKAP11*, *ARHGAP35*, *BMP7*, *GPC3*, *NEK1*, *PMEPA1*, *ROBO1*, *RSPO2*, *SLC26A7*) were significantly enriched in phenotype categories including “abnormal kidney collecting duct morphology,” “increased urine osmolality,” “kidney cortex cysts,” “kidney cysts,” “polycystic kidney,” “hydroureter,” and “abnormal kidney development” (Additional file 1: Table s17). Eleven PSGs (*ADCYAP1R1*, *ATF4*, *FTO*, *GHRHR*, *MYO7A*, *OCA2*, *PDE10A*, *PDE3B*, *PMCH*, *TNKS*, *UNC5C*) were significantly enriched in phenotype categories related to energy intake, including “increased insulin secretion,” “increased circulating insulin level,” “increased food intake,” and “decreased circulating leptin level” (Additional file 1: Table s17). Taken together, extensive natural selection of genes associated with osmoregulation and energy homeostasis implies that water and food deprivation have likely been the main drivers behind the evolution of arid desert adaptation in hairy-footed jerboas.

## Discussion

### Genomic evidence supports three species in hairy-footed jerboas

The intraspecific taxonomy of hairy-footed jerboas (*Dipus*) has not been well resolved because of previous limited molecular coverage and some variable morphological traits, such as pelage color, which vary significantly with season and age [54]. The most recent taxonomic assessment conservatively divided the hairy-footed jerboas into two species, *D. deasyi* and *D. sagitta* [12]. Due to insufficient genetic evidence, the latter was treated as a species complex including three groups: turanicus (distributed outside China), sagitta, and sowerbyi [12]. Here, based on multiple genetic evidence (i.e., genome-wide SNPs, mitochondrial genomes, different demographic historical trajectories, and species delimitation results), we clearly revealed three significant divergent genetic lineages within the genus *Dipus* in China’s arid zone. These findings confirm the taxonomic status of *D. deasyi* and support the recognition of *D.sagitta* and *D. sowerbyi* as valid species. This classification is further supported by prior morphological evidence. For example, Cheng et al. [12] showed that the three species can be readily differentiated based on skull measurements and overall cranial shape. In particular, *D. sagitta* has the largest size and the widest postorbital constriction and mastoid breadth, but the smallest palatine foramen. *D. deasyi* has the largest appendages (tail and ear), largest tympanic bulla and mandibular toothrow, and the narrowest postorbital constriction, and *D. sowerbyi* has the smallest hind foot [12]. Therefore, by combining prior taxonomic assessment and systematic studies [11, 12, 55] with our genomic results, we propose that the hairy-footed jerboas in China’s arid zone are composed of three species rather than two, namely *D. deasyi*, *D. sagitta*, and *D. sowerbyi*.

The distribution boundaries of *D. sagitta* and *D. sowerbyi* are delimited by two huge mountain ranges, the Tianshan Mountains and the Altay Mountains in northwest China (Fig. 1A, B), which is consistent with prior studies [55]. Additionally, the Tianshan Mountains are also the dispersal barrier between *D. deasyi* and *D. sagitta* (Fig. 1A, B). For *D. deasyi* and *D. sowerbyi*, the most likely geographical barriers are the salt lakes and saline wastelands located in the easternmost side of the Tarim Basin and the central Qaidam Basin, similar to the Chott el Djerid salt lake barrier for the greater Egyptian jerboa, *Jaculus orientalis* [56].

### Positive selection of ATR and HIF1AN promotes high-altitude desert adaptation

UV has been considered to be one of the important environmental genotoxic agents for organisms [57], especially in high-altitude regions, as UV exposure increases by about 10% for every 1000-m increase in elevation [58, 59]. To successfully survive in high-altitude environments, species need to cope with enhanced UV conditions. UV irradiation induces the formation of covalent thymine dimers between successive bases, which breaks DNA structure and causes DNA damage [60]. Unrepaired DNA damage leads to genomic instability and has genotoxic and cytotoxic effects on cells, thus leading to deterioration of cell function and even cell death [61, 62]. The GO enrichment analysis of genes associated with selective sweeps in high-altitude desert group showed that five PSGs (*ATR*, *INIP*, *MCM8*, *MEF6*, *RAD51*) were observably enriched in GO terms associated with DNA repair system, such as DNA double-strand break repair and recombinational repair (Additional file 1: Table s9). At the cellular level, the repair system can replace damaged DNA and thus increase cell adaptation to UV stress [61–63]. Therefore, positive selection on the DNA repair system might help hairy-footed jerboas adapt to the intense ultraviolet environment at high altitudes. Furthermore, among the five genes, *ATR*, which harbored seven missense candidate SNPs, showed the strongest selective sweep in the genome (Fig. 3C–E). *ATR* has been implicated in the response to UV and controls the cell-cycle after DNA damage through the p53 signaling pathway [64, 65]. The p53 pathway has been dubbed the guardian of the genome because of its ability to protect the cell by responding to cellular insults by inducing cell cycle arrest or apoptosis [66, 67]. *ATR* is located in the upstream of p53 pathway. After UV, the *ATR* and ATR kinase (*CHK1*) are induced, which will increase the levels and activity of p53 protein and activate DNA repair systems in the p53 downstream pathway, thereby protecting the genome integrity and avoiding cell apoptosis [67, 68]. Seven alleles in the *ATR* gene coding region showed significant gene frequency differences between high-altitude desert group and low-altitude desert group (Fig. 3E), which was potentially associated with UV stress, indicating a causal mutation contributing to high-altitude adaptation.

Hypoxia is also an important environmental stress at high altitudes. As a master regulator of the cellular response to hypoxia, *HIF-1α* activates transcription of genes whose protein products either increase oxygen availability by promoting erythropoiesis and angiogenesis or mediate the adaptive responses to oxygen deprivation, such as increased glycolytic metabolism [69–71]. Previous comparative genomics studies had shown that many genes involved in the HIF-1 signaling pathway were considered candidate adaptive elements for hypoxia adaptation in several species and that adaptations varied from species to species in different regions [72–77]. In our result, we observed that a PSG, *HIF1AN*, involved in the HIF-1 signaling pathway, was strongly positively selected in the high-altitude population. *HIF1AN* is a well-known negative modulator of *HIF-1α*. The frequency of occurrence of the *HIF1AN* alleles was conspicuously different in high-altitude desert group and low-altitude desert group of hairy-footed jerboas (Additional file 3: Fig. s7), indicating that this gene played a possible role in high-altitude adaptation.

### Water and food deprivation are the main drivers of evolution in deserts.

Food and water availability are very important for all organisms living in deserts. When food is scarce, metabolism is completely dependent on endogenous nutrients, such as sugars, fats, and proteins. Nutrient homeostasis adaptation is considered to be one of the major shared mechanisms of arid desert adaptation across mammals [3, 7–9, 78, 79]. Our results found that the hairy-footed jerboa populations in different types of deserts exhibited significant positive selection in these genes associated with energy, glucose, fat, or protein metabolism. Specifically, the high-altitude desert group showed strong positive selection mainly in fat homeostasis and metabolism, the hyper-arid desert group showed positive selection mainly in glucose metabolism, protein metabolism, and insulin signaling, while the arid desert group showed obvious positive selection in glucose, protein, and fat metabolism and increased energy intake (Additional file 1: Table s18). Fat metabolism involves adaptive tolerance not only to food shortages but also to heat/cold and dehydration [3, 80, 81]. It had been found that the enhancement of fat metabolism could enhance the thermogenic performance of high-altitude adapted deer mice [82]. Therefore, positive selection of genes related to fat metabolism in the high-altitude desert group may contribute to adaptation to cold and food scarcity in high-altitude desert. Interestingly, a higher proportion of PSGs was detected in both the hyper-arid desert group and arid desert group involved in protein metabolism compared to glucose and fat metabolism (Additional file 1: Table s18). It is well known that the energy return of protein catabolism is significantly lower than that of lipid catabolism, but the amount of metabolic water produced is more than five times that of lipid metabolism [83, 84]. Physiological experiments have also confirmed that laboratory mice do increase the rate of protein catabolism during dehydration [83]. Positive selection in protein metabolism may be an important water retention strategy of hairy-footed jerboas to adapt to arid deserts, which is consistent with the protein-for-water hypothesis [83–85].

Moreover, osmoregulatory homeostasis is the key mechanism to maintain proper water and electrolyte balance, especially in water-deprivation conditions. Previous studies have found that the osmoregulatory mechanism of rodents in desert environments with water shortage is often under selection [86, 87]. Similarly, we observed 7 genes (*ANK2*, *CALB1*, *CLCN3*, *DMXL2*, *HCRTR2*, *ITPR1*, and *SLC24A5*) involved in cellular homeostasis from the hyper-arid desert group and 6 genes (*ADCYAP1R1*, *ATF4*, *CACNA1I*, *ITPR1*, *Nr0b1*, and *ITPR1*) involved in renin secretion and cortisol synthesis and secretion pathways from the arid desert group experienced a significant selective sweep (Additional file 1: Table s18), suggesting the importance of osmoregulation in desert adaptation. Additionally, as dehydration reduces cell volume and causes cytoskeletal rearrangement [88], the top over-represented actin cytoskeleton in the arid desert group may also indicate an additional adaptation to water scarcity in the hairy-footed jerboas (Additional file 1: Table s15). Besides, the kidney is the main organ that regulates the balance of water and osmotic pressure in the body. Prolonged dehydration causes the body to produce urine with higher concentration of minerals and waste, which can lead to the formation of crystals that can affect kidney function and even cause kidney lesions [89]. Phenotypic enrichment analysis found that 9 PSGs (*AKAP11*, *ARHGAP35*, *BMP7*, *GPC3*, *NEK1*, *PMEPA1*, *ROBO1*, *RSPO2*, and *SLC26A7*) were closely associated with increased urine osmolality, hydroureter, polycystic kidney, and kidney development. These findings may provide more evidence for the hairy-footed jerboas’ adaptation to water-scarcity.

## Conclusions

Population genomic analyses of hairy-footed jerboas support three species in China, *D. deasyi*, *D. sagitta*, and *D. sowerbyi*, with no gene flow among them. Comparative genomic analyses of *D. sowerbyi* populations across different desert environments revealed a variety of candidate loci that may underlie local adaptations to distinct desert conditions. Specifically, the stronger signatures of selection in gene *ATR* and *HIF1AN* are consistent with adaptations under intense UV radiation and hypoxia in high-altitude desert animals, and the genes involved in energy and water homeostasis were a pervasive signature of selection in hyper-arid and arid desert environments. The functional and phenotypic enrichment of PSGs indicated that the genetic basis of energy and water homeostasis adaptation traits in desert may be highly polygenic. These results contribute to our understanding of the genetic mechanism of rapid radiation adaptation of hairy-footed jerboa species to diverse desert environments and provide information for future evolutionary and physiological genomics research.

## Methods

### Sample collection, sequencing, and mitochondrial genome assembly

A total of 83 hairy-footed jerboa samples were collected for genome sequencing from eight desert zones, including the Horqin Sandy Land (HRQ, *n* = 9), Gobi Desert (GOB, *n* = 23), Mu Us Desert (MUS, *n* = 8), Junggar Basin (JGR, *n* = 9), Bosten Lake Region (BST, *n* = 8), the eastern of Qaidam Basin (EQM, *n* = 9), the western of Qaidam Basin (WQM, *n* = 7), and Tarim Basin (TRM, *n* = 10), covering almost all geographical populations of hairy-footed jerboa in China (Fig. 1A; Additional file 1: Table s1). In addition, we also collected a long-eared jerboa (*Euchoreutes naso*, Euchoreutinae, Dipodidae) from the northwestern Tarim Basin and used it as a phylogenetic outgroup (Additional file 1: Table s3). The genomic DNA of all samples was extracted using a Qiagen DNA purification kit (Qiagen, Valencia, CA, USA) following its standard protocol. At least 1 μg of genomic DNA per sample was used to construct a library with an insert size of ~150bp and then sequenced using the Illumina HiSeq 2000 sequencing platform. Meanwhile, all the clean reads for each individual were used for de novo assembling a mitochondrial genome by GetOrganelle v.1.7.6 [90]. Then, we checked the assembly graph to verify the exported complete mitogenome by Bandage [91].

### Variant calling and annotation

The clean reads were aligned against the Sowerby’s three-toed jerboa reference genome from Tianshan, Inner Mongolia, China, generated in our previous study (NCBI accession number: PRJNA682294) [10, 92] using Burrows-Wheeler Aligner v.0.7.17 (BWA-mem; [93]) software with the default parameters. SAM files were firstly converted and sorted by Samtools v.1.14 [94] and then marked as PCR duplicates with Picard v.2.26.6 (https://broadinstitute.github.io/picard/). After that, Bcftools v.1.14 [95] was used to identify SNPs (single nucleotide polymorphisms) and InDels (short insertions and deletions) with all individuals simultaneously. To obtain reliable candidate variant set, the merged raw variant set was filtered by VCFtools v.0.1.16 [96] using the command parameters: “--mac 3 --maf 0.05 --minQ 30 --minDP 4 --maxDP 50 --thin 5 --min-alleles 2 --max-alleles 2 --max-missing 0.2”. After filtering, the remaining high-quality variant set was split into SNPs set and InDels set and then was annotated according to the GFF file of the Sowerby’s three-toed jerboa reference genome using SnpEff v.4.3 [22].

### Population structure analysis

After filtering, we obtained a set of high-quality SNPs for the following population structure analyses. Firstly, we used the Treebest v.1.9.2 [97] to construct the individual-based NJ tree for all samples based on the nucleotide *p-*distance matrix. The NJ tree was rooted using the long-eared jerboa (*E. naso*) as outgroup and visualized with iTOL (http://itol.embl.de/). Then, PCA of whole-genome SNPs for all 83 individuals was performed with the GCTA v.1.93.2 [98]. Furthermore, ancestry and population structure analysis were performed on all 83 hairy-footed jerboas and on the 57 *D. sowerbyi* individuals using sNMF v1.2 [99] with the default parameters. To reduce computing time, an LD pruning step was performed with Plink v.1.9 [100] with the following parameters: “--indep-pairwise 50 10 0.1”. The number of assumed genetic clusters K ranged from 1 to 10. The optimal K was determined by the minimal value of the cross-entropy criterion of sNMF. In addition, we also performed the phylogenetic analysis with the mitochondrial genomes data. A NJ tree was constructed by using MEGA [101] with 1000 bootstrap replicates.

### *Population genetic divergence (F*_*ST*_*) and species delimitation*

We calculated the genome-wide distribution of *F*_ST_ values among the three hairy-footed jerboa species by using the VCFtools v.0.1.16 [96] with a sliding-window method (100-kb window with 50-kb increment). Then, we ran the species delimitation analysis for hairy-footed jerboas with the SNAPP package (v.1.5.5) in BEAST v.2.6 [102]. To reduce the computational load and the influence of sample size bias, SNPs of three individuals from each of the twelve geographical locations (totaling 36 individuals) were randomly selected, and the VCFtools [96] with parameters “--max-missing 1” was used to remove the missing data. The final SNP data was then used for the following SNAPP analysis. We calculated the marginal likelihood estimate (MLE) and Bayes factor (BF) for each possible species delimitation model (Additional file 1: Table s5) with the setting “chainLength = 100,000 preBurnin = 10,000 nrOfSteps = 24” for the stepping-stone analysis in SNAPP. All BF were made against the first model (RunA), and negative BF values indicate support for the first model (RunA). The BF scale is as follows: 0 < BF < 2 is not worth more than a bare mention, 2 < BF < 6 is positive evidence, 6 < BF < 10 is strong support, and BF > 10 is decisive.

### Demographic history and gene flow

To better understand historical changes in ancestral hairy-footed jerboas, the PSMC [103] software was used to estimate the changes in the effective population size (*N*_*e*_) over the last one million years. The PSMC parameters were set as follows: -N30 -t15 -r5 -p “4+25*2+4+6”. The generation time (g) was set to 1.5 years, and the average mutation rate (μ) was set to 6.319 × 10^−9^ per base per generation [10]. To avoid biased estimation [104], the individual with the highest sequencing depth (above 18×) from each genetic lineage (TRM10, JGR9, BDJ4) was selected for PSMC analysis (Additional file 1: Table s2). To test the gene flow among three hairy-footed jerboa species, we used f3-statistics in Admixtools v.7.02 [35]. We also used Treemix v.1.13 [36] to detect the gene flow and its directions among species. The program generated the maximum likelihood (ML) tree and a corresponding residuals matrix for the three species, and the matrix was used to identify the population pairs with poor fit in the ML tree.

### Genome-wide selective sweep test and candidate gene analysis

The above phylogenetic analysis and population structure analysis divided the hairy-footed jerboas of China into three species, *D. deasyi*, *D. sagitta*, and *D. sowerbyi*, and the latter occupies a more diverse desert environment. To identify genomic regions influenced by local adaptation in *D. sowerbyi*, we compared the genomes for three extreme-control group pairs, which include the high-altitude desert group (QDME1-9) versus low-altitude desert group (HRQ1-9), the hyper-arid desert group (EJN1-3, YWC1-3) versus semi-desert group (HRQ1-9, MWS1-8, QDME4-6), and the arid desert group (BDJ1-6, HTP1-3, HSD1-8, EJN1-3, YWC1-3, BST1-8, QDME1-3, QDME7-9) versus semi-desert group (HRQ1-9, MWS1-8, QDME4-6) (Additional file 1: Table s2). We calculated the population *F*_ST_ value and *π*-ratios (i.e., *π*_*-*Low-altitude Desert/High-altitude Desert_, *π*_*-*Semi-desert/Hyper-arid Desert_, and *π*_*-*Semi-desert/Arid desert_) across the genome using a sliding method (100-kb windows and 50kb steps). Only the windows with the top 5% of *F*_ST_ value and *π*-ratios simultaneously were considered as the candidate outliers under strong selective sweeps. Then, those outlier windows were assigned to corresponding SNPs and genes. The GO and functional pathway analyses of candidate genes were implemented with the online tool Metascape [105]; *P* values were calculated based on the cumulative hypergeometric distribution. The phenotype enrichment analysis was conducted based on phenotype data from Mouse Genome Informatics (MGI) Mammalian Phenotype data using the web application Enrichr [106]. Functional information for each candidate gene was consulted based on the annotations in the NCBI (http://www.ncbi.nlm.nih.gov, last accessed January 2, 2023) and SwissProt (http://www.uniprot.org, last accessed January 2, 2023) databases.

### Supplementary Information

**Additional file 1:** **Table s1.** Sampling information of 83 *Dipus* jerboas. **Table s2.** Definition of the environment types and the *Dipus* individuals living in these environments. **Table s3.** Sequencing information of 83 *Dipus* samples and one long-eared jerboa, *Euchoreutes naso*. **Table s4. **Summary information for SnpEff annotation of *Dipus* population genomic variants. **Table s5. **Pairwise *F*_ST_ values among the three *Dipus* species and eight populations. **Table s6. **Summary of f3-statistic. **Table s7. **Path sampling results for species delimitation analyses. **Table s8.** Genes under positive selection in *D. sowerbyi* from high altitude deserts.** Table s9.** GO enrichment result of PSGs in *D. sowerbyi *individuals from high-altitude deserts. **Table s10.** Allele frequency statistics of seven missense mutations of* ATR* gene. **Table s11. **Genes under positive selection in *D. sowerbyi* from hyper-arid deserts. **Table s12. **GO enrichment result of PSGs in *D. sowerbyi* individuals from hyper-arid deserts. **Table s13. **Allele frequency statistics of six missense mutations of *Dmxl2* gene. **Table s14.** Genes under positive selection in *D. sowerbyi *from arid deserts. **Table s15.** GO enrichment result of PSGs in *D. sowerbyi *individuals from arid deserts. **Table s16. **KEGG pathways enrichment result of PSGs in *D. sowerbyi* individuals from arid desert. **Table s17. **Mouse phenotype enrichment of 203 PSGs in *D. sowerbyi *from arid deserts. **Table s18. **Summary of the GO categories of the candidate genes associated with energy homeostasis and hypertonicity adaptation.

**Additional file 2. **The mitochondrial genome of 83 hairy-footed Jerboas.

**Additional file 3: Fig. s1.** Phylogenetic neighbor joining tree of whole-genome SNPs (83 *Dipus *individuals) with long-eared jerboa (*Euchoreutes naso*) as outgroup. **Fig s2.** NJ tree of all 83 *Dipus* jerboas based on mitochondrial genomes sequences with a long-eared jerboas (*Euchoreutes naso*) as outgroup. **Fig s3.** Principal component analysis (PCA) of the all 83 *Dipus* jerboas. **Fig. s4.** Population structure analysis of the all 83 *Dipus* jerboas by using sNMF. **Fig. s5.** The cross-validation error rate of different K values for all 83 *Dipus* jerboas and 57 *D. sowerbyi* jerboas in sNMF analysis. **Fig. s6. **The maximum-likelihood tree and residuals generated by TreeMix with no inter-group migration. **Fig. s7.** Variation in the *HIF1AN* gene among two groups of *Dipus* jerboas.

## Data Availability

The genome sequencing data in the present study have been deposited in NCBI under projection accession number PRJNA931436 [92]. The hairy-footed jerboa reference genome analyzed was generated in our previous study (NCBI accession number: PRJNA682294; [10, 21]). The final filtered SNP/InDel set used for the analyses in the present study has been deposited in figshare (https://doi.org/10.6084/m9.figshare.23802876; [107]).

## Abbreviations

HRQ: Horqin Sandy Land
GOB: Gobi Desert
MUS: Mu Us Desert
BST: Bosten Lake region
EQM: The eastern of Qaidam Basin
WQM: The western of Qaidam Basin
JGR: Junggar Basin
TRM: Tarim Basin
SNP: Single nucleotide polymorphism
InDels: Insertion–deletion mutations
NJ: Neighbor-joining
ML: Maximum likelihood
PCA: Principal component analysis
Mya: Million years ago
GO: Gene ontology
KEGG: Kyoto encyclopedia of genes and genomes
UV: Ultraviolet radiation
PSGs: Positive selection genes
