# Supplementary material for: Whole-genome sequencing reveals adaptations of hairy-footed jerboas (Dipus, Dipodidae) to diverse desert environments
Source: BMC Biol. 2023 Aug 30;21:182. doi: 10.1186/s12915-023-01680-5 (PMC10469962; doi:10.1186/s12915-023-01680-5)
Supplement: Supplementary file 3 — Additional file 3: Fig. s1. Phylogenetic neighbor joining tree of whole-genome SNPs (83 Dipus individuals) with long-eared jerboa (Euchoreutes naso) as outgroup. Fig s2. NJ tree of all 83 Dipus jerboas based on mitochondrial genomes sequences with a long-eared jerboas (Euchoreutes naso) as outgroup. Fig s3. Principal component analysis (PCA) of the all 83 Dipus jerboas. Fig. s4. Population structure analysis of the all 83 Dipus jerboas by using sNMF. Fig. s5. The cross-validation error rate of different K values for all 83 Dipus jerboas and 57 D. sowerbyi jerboas in sNMF analysis. Fig. s6. The maximum-likelihood tree and residuals generated by TreeMix with no inter-group migration. Fig. s7. Variation in the HIF1AN gene among two groups of Dipus jerboas. [file 12915_2023_1680_MOESM3_ESM.docx]

Supplementary Materials for

**Whole-genome sequencing reveals adaptations of hair-footed jerboas (*Dipus* Dipodidae) to diverse desert environments**

Xingwen Peng^1, 2, †^, Jilong Cheng^1, †^, Hong Li^3^, Anderson Feijó^1,4^, Lin Xia^1^, Zhixin Wen^1^, Deyan Ge^1^, Qisen Yang^1, *^

^∗^Corresponding author:

**Qisen Yang**

E-mail: yangqs@ioz.ac.cn, +86-10-64807225.

**This PDF file includes:**

Supplementary Fig. s1-s7

**
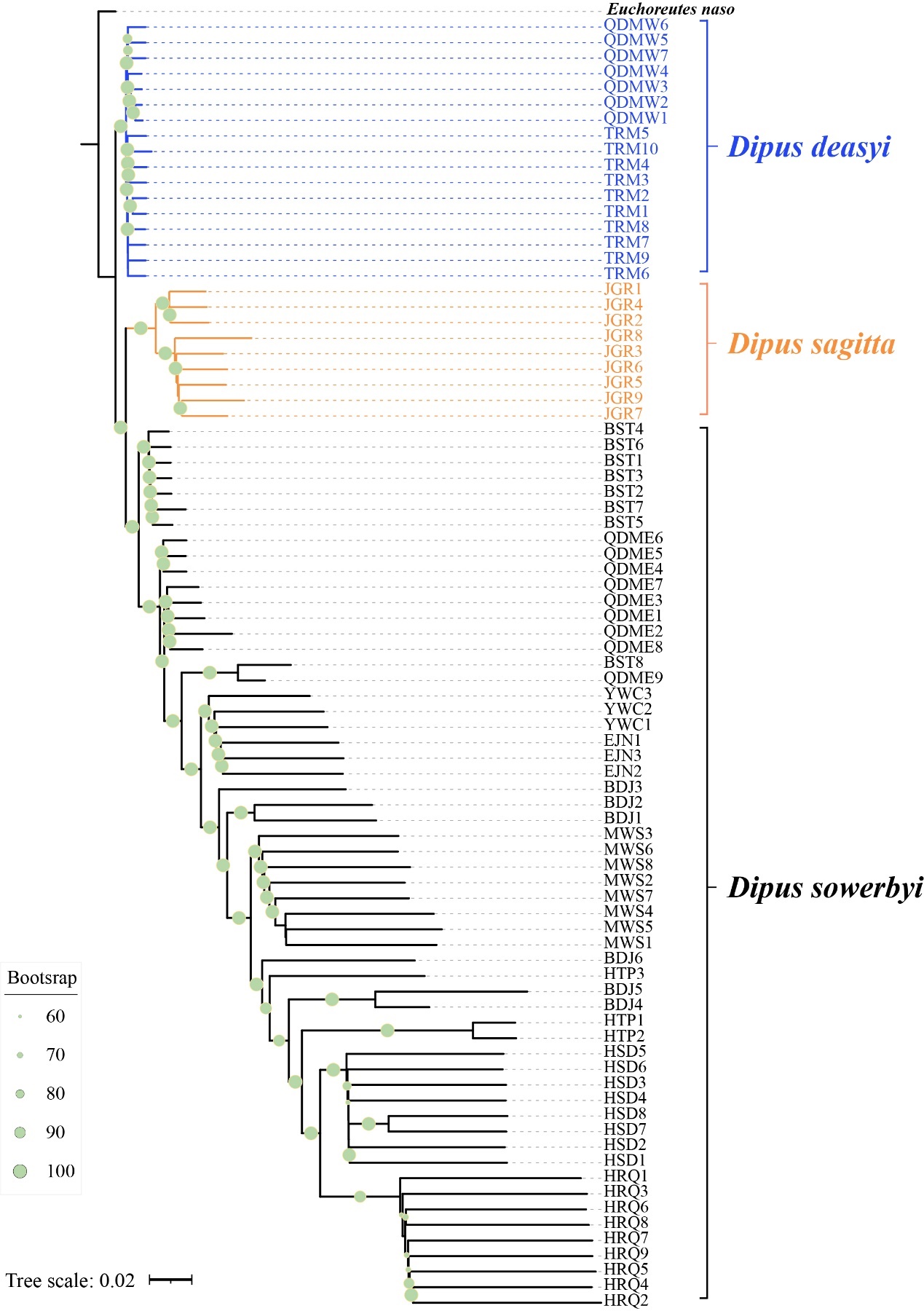
**

**Fig. s1. Phylogenetic neighbor joining tree of whole-genome SNPs (83 *Dipus* individuals) with long-eared jerboa (*Euchoreutes naso*) as outgroup.** For the abbreviations of the individuals, see supplementary table s1; Supplementary Material online.

**
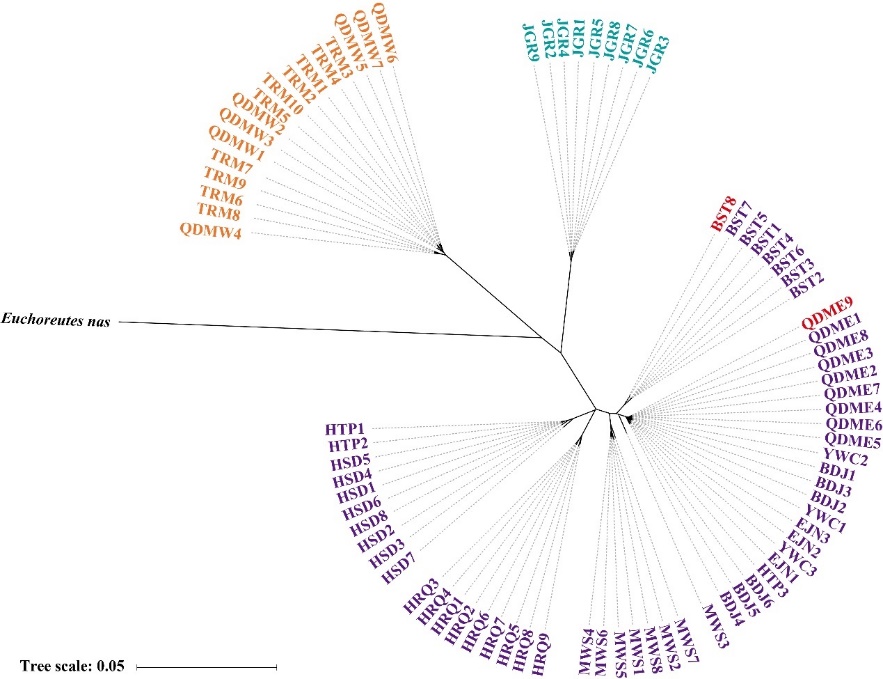
**

**Fig. s2. NJ tree of all 83 Dipus jerboas based on mitochondrial genomes sequences with a long-eared jerboas (*Euchoreutes naso*) as outgroup.** For the abbreviations of the individuals, see supplementary table s1; Supplementary Material online.


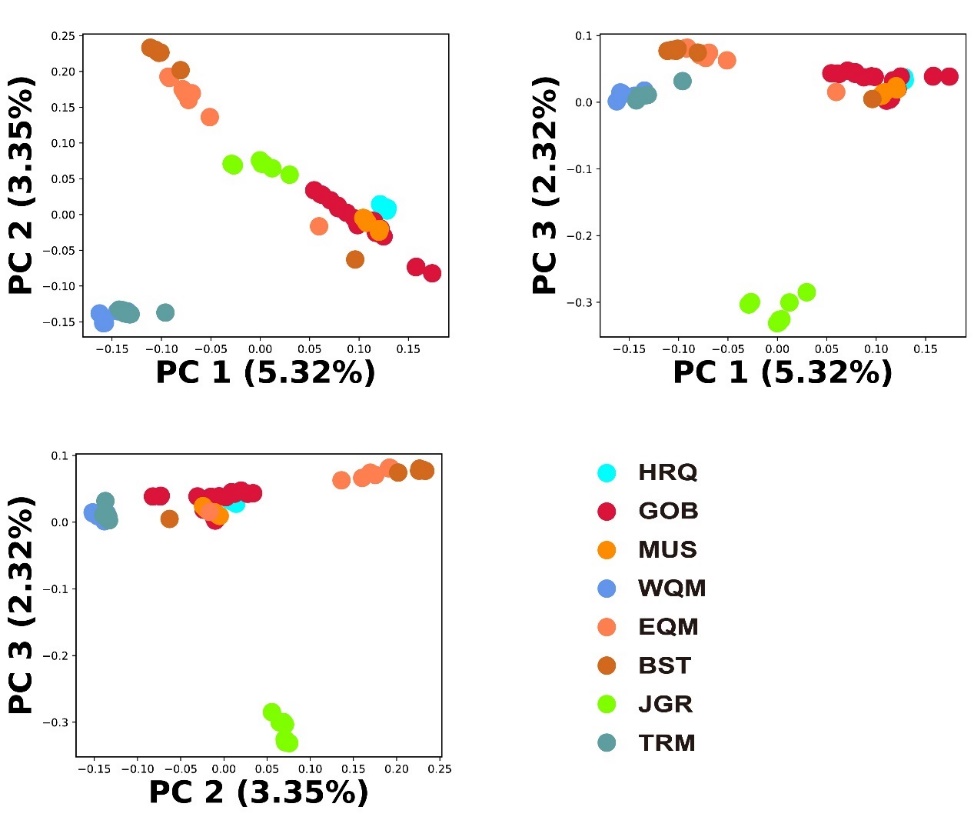


**Fig. s3. Principal component analysis (PCA) of the all 83 Dipus jerboas.** Different colors represent individuals from different sampling region. Five dotted circles indicate the five populations (DEA, Tarim-Western Qaidam population; JGR, Junggar Basin population; BST, Bosten Lake population; QDM, Eastern-Qaidam population; ENC, Eastern-Northern China population). **
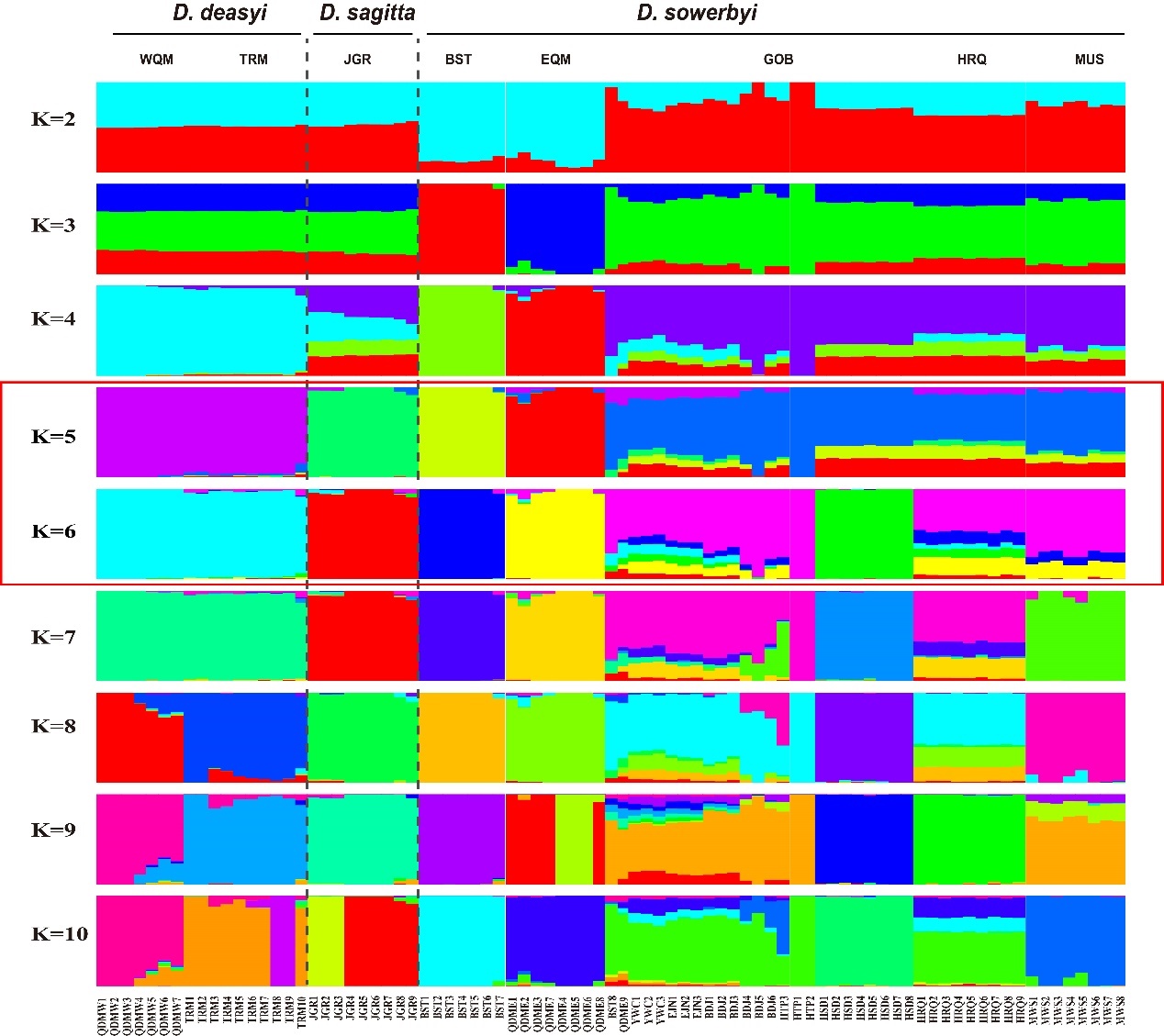
**

**Fig. s4. Population structure analysis of the all 83 Dipus jerboas by using sNMF.** The length of each colored segment represents the proportion of the individual’s genome inferred from K = 2-10 ancestral populations. For the abbreviations of the individuals, see supplementary table s1; Supplementary Material online.


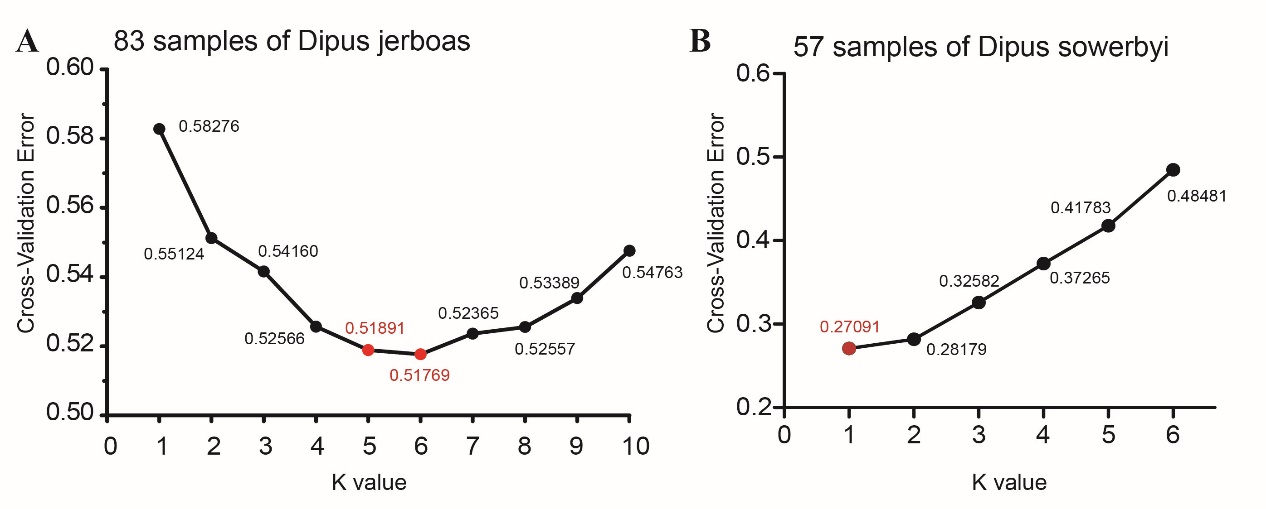


**Fig. s5. The cross-validation (CV) error rate of different K values (from 1 to 10) for all 83 *Dipus* jerboas and 57 *D. sowerbyi* jerboas in sNMF analysis.**

**
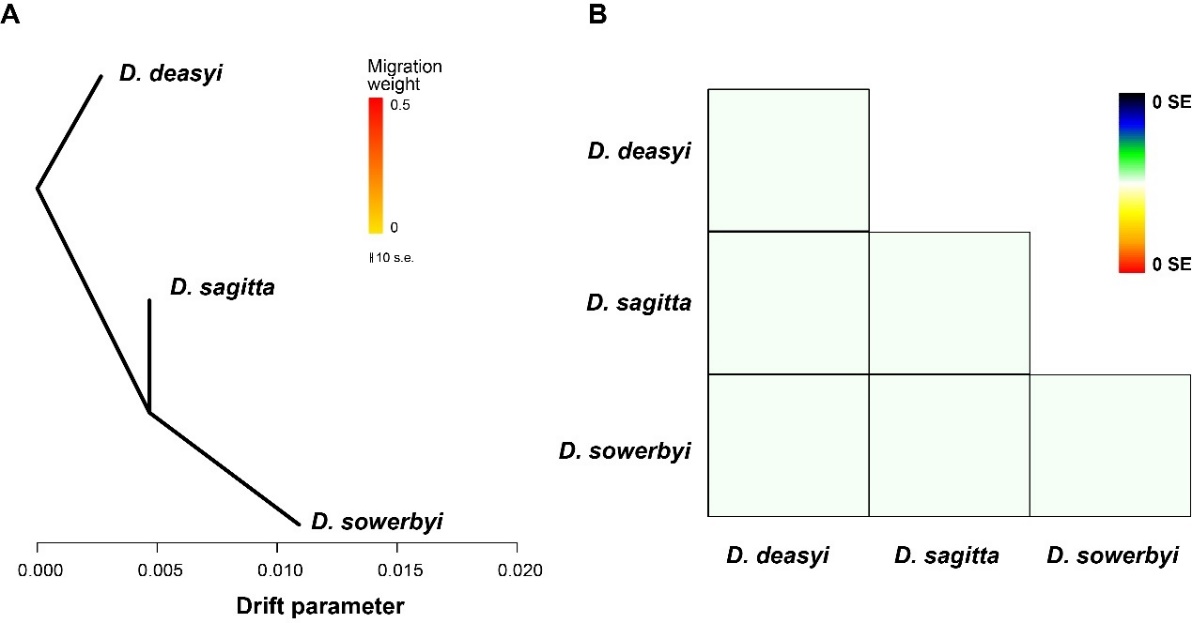
**

**Fig. s6. A) The maximum-likelihood tree generated by TreeMix with no inter-group migration.** The scale bar shows ten times the average standard error of the entries in the sample covariance matrix. **B) Residuals of the maximum-likelihood phylogenetic tree.** As implemented in TreeMix, the residual covariance between each pair of populations i and j is divided by the average standard error across all pairs. This scaled residual in then plotted in each cell (i,j). Colours are described in the palette on the right. Residuals trend zero represent no admixture events between populations (SE: standard error).


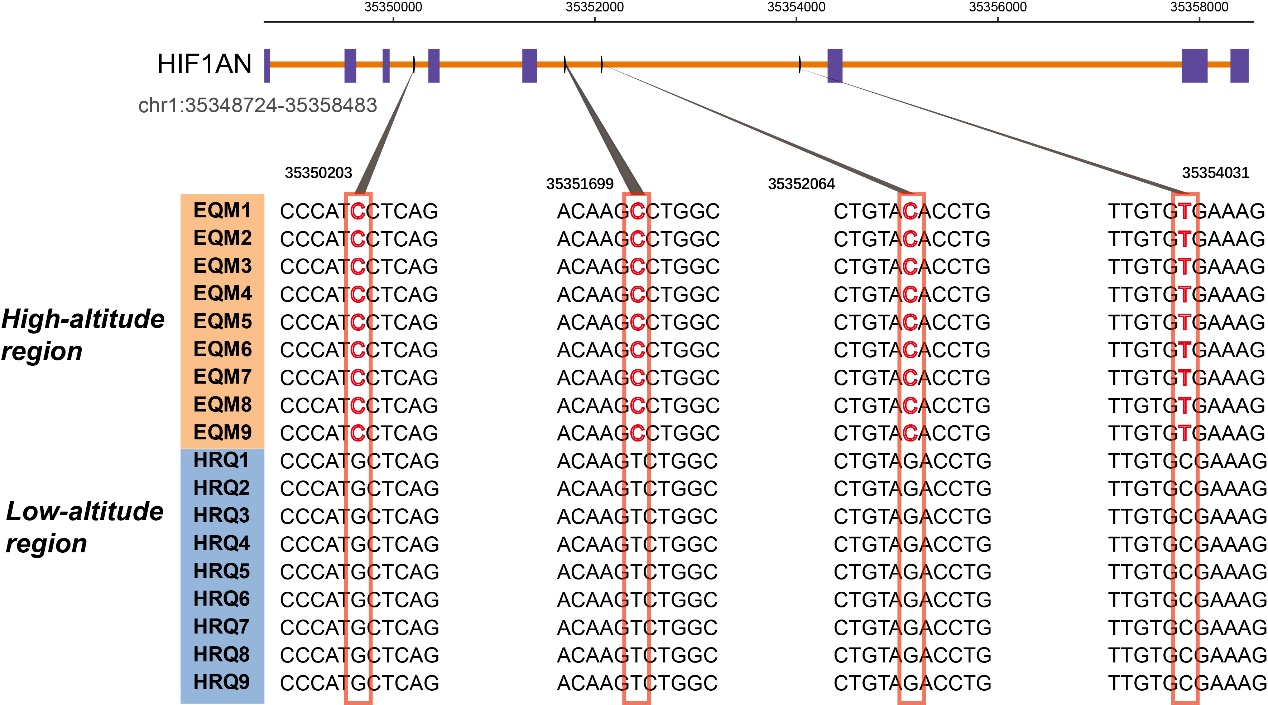


**Fig. s7. Variation in the HIF1AN gene among two groups of *Dipus* jerboas.** Four nucleotide replacements in High-altitude arid group compared with the Low-altitude arid group of *Dipus* jerboas are shown in red and marked with boxes.
